# DNA-Fragments Are Transcytosed across CaCo-2 Cells by Adsorptive Endocytosis and Vesicular Mediated Transport

**DOI:** 10.1371/journal.pone.0056671

**Published:** 2013-02-11

**Authors:** Lene E. Johannessen, Bjørn Spilsberg, Christer R. Wiik-Nielsen, Anja B. Kristoffersen, Arne Holst-Jensen, Knut G. Berdal

**Affiliations:** Norwegian Veterinary Institute, Oslo, Norway; Ecole Polytechnique Federale de Lausanne, Switzerland

## Abstract

Dietary DNA is degraded into shorter DNA-fragments and single nucleosides in the gastrointestinal tract. Dietary DNA is mainly taken up as single nucleosides and bases, but even dietary DNA-fragments of up to a few hundred bp are able to cross the intestinal barrier and enter the blood stream. The molecular mechanisms behind transport of DNA-fragments across the intestine and the effects of this transport on the organism are currently unknown. Here we investigate the transport of DNA-fragments across the intestinal barrier, focusing on transport mechanisms and rates. The human intestinal epithelial cell line CaCo-2 was used as a model. As DNA material a PCR-fragment of 633 bp was used and quantitative real time PCR was used as detection method. DNA-fragments were found to be transported across polarized CaCo-2 cells in the apical to basolateral direction (AB). After 90 min the difference in directionality AB vs. BA was >10^3^ fold. Even undegraded DNA-fragments of 633 bp could be detected in the basolateral receiver compartment at this time point. Transport of DNA-fragments was sensitive to low temperature and inhibition of endosomal acidification. DNA-transport across CaCo-2 cells was not competed out with oligodeoxynucleotides, fucoidan, heparin, heparan sulphate and dextrane sulphate, while linearized plasmid DNA, on the other hand, reduced transcytosis of DNA-fragments by a factor of approximately 2. Our findings therefore suggest that vesicular transport is mediating transcytosis of dietary DNA-fragments across intestinal cells and that DNA binding proteins are involved in this process. If we extrapolate our findings to *in vivo* conditions it could be hypothesized that this transport mechanism has a function in the immune system.

## Introduction

Although most of the dietary DNA that is consumed by an organism is degraded to nucleosides and bases before being absorbed in the gastrointestinal tract, we and others have shown that up to 1% of dietary DNAs are able to pass the intestinal barrier as longer fragments and enter the blood stream of humans [1], chicken [2], pig [3], mice [4] and fish [5], [6] These fragments are thereafter transported to different organs before they eventually become degraded. DNA-fragments of up to 300 bp in size have been reported in organs of Atlantic salmon after force feeding [6] and in humans [1]. The observed transport of DNA across the intestinal barrier has been suggested to have an immunomodulatory role [7]. In the human intestinal cell line CaCo-2 transcellular transport of single stranded short oligodeoxynucleotides (ODNs) has been suggested [8].

Several DNA-binding proteins have been isolated from cellular membranes [9], although involvement of these proteins in uptake of longer dsDNA is uncertain. Toll like receptor 9 (TLR9) [10] is a pattern recognition receptor (PRR) recognizing different pathogens by identifying molecular patterns (PAMPs) found on bacteria. It acts by binding unmethylated CpG motifs. In enterocytes the TLR9 is localized at the plasma membrane (PM) both apically and basolaterally where it is involved in maintenance of colonic homeostasis [11]. Activation of PRRs by PAMPs activate the innate immune system. DNA with phosphothioate backbone activates TLR9 only when containing unmethylated CpG DNA motifs. Other PRRs localized at the PM have also been suggested to be able to recognize DNA. These include different members of the Scavenger Receptor (SR) family, namely Macrophage Receptor with Collagenous structure (MARCO), Chemokine (C-X-C motif) ligand 16 (CXCL16) and Stabilin 1/2 [12], [13]. A nucleic acid channel (NACh) capable of mediating uptake of single stranded nucleic acids was demonstrated in rat renal brush-border membranes and brain [14], [15]. Nucleolin, a DNA and RNA binding nuclear shuttling protein, is also present at the cell surface and is able to mediate uptake of DNA [16].

In order to characterize transport of DNA-fragments across the intestine, we used the human intestinal epithelial cell line CaCo-2 as a model. These cells polarize and differentiate into absorptive enterocytes upon growth on filters. As dietary DNA we used a 633 bp long polymerase chain reaction (PCR) amplified fragment. We have previously used similar PCR-fragments to study DNA uptake from the diet in Atlantic salmon (*Salmo salar*) [6]. Using this PCR-fragment as DNA material, we found that DNA-fragments were transcytosed by vesicular mediated transport in the apical to basolateral direction in CaCo-2 cells, and that DNA-binding proteins other than TLR9, SR and heparin binding proteins seem to be involved in this process. If we extrapolate our data to *in vivo* conditions over a human/animal lifespan and evolutionary time, it can be expected that transcytosis of DNA can have a significant biological role possibly modulating immune responses.

## Results

### Time-course of DNA-fragment transport across polarized CaCo-2 cells

**Figure 1:**
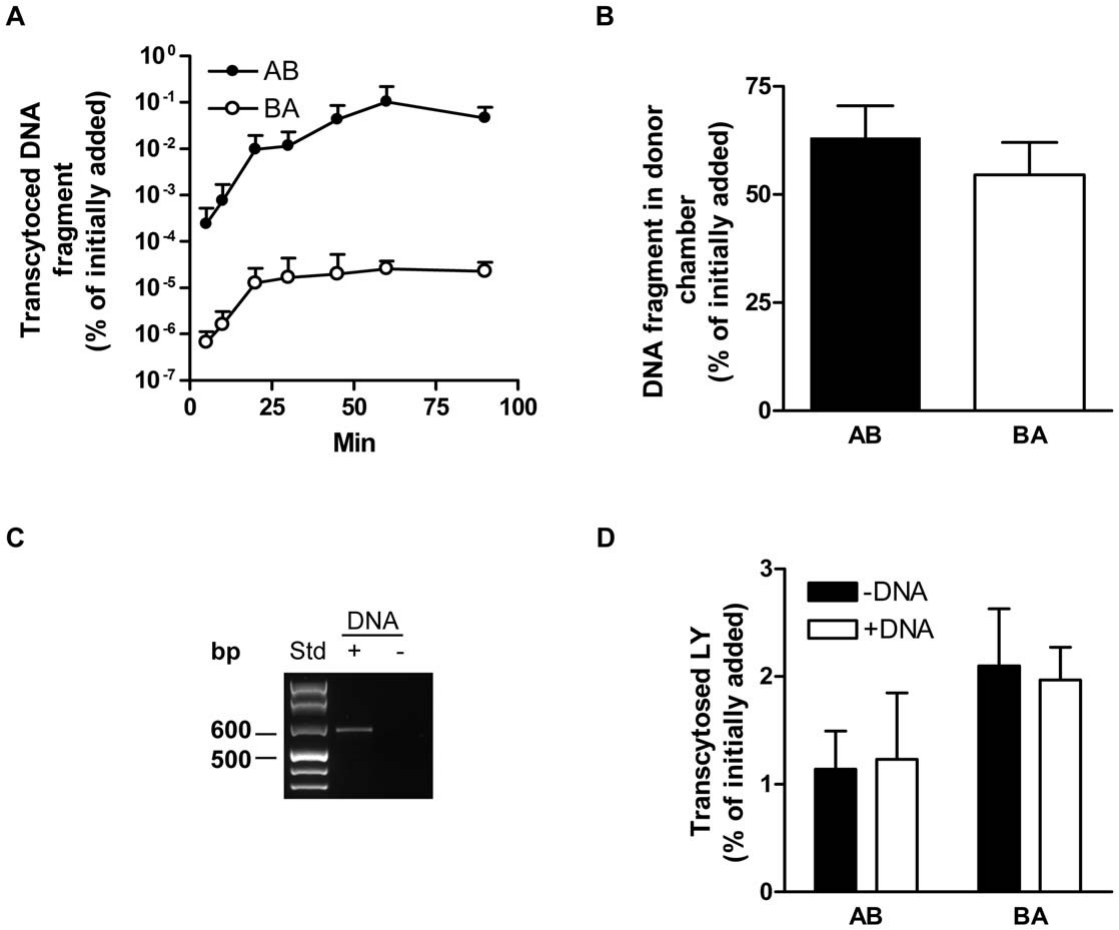
Time-course of DNA-fragment transport across CaCo-2 cells. CaCo-2 cells on filters were incubated with a 633 bp long polymerase chain reaction (PCR) amplified fragment and Lucifer yellow (LY) at 37 °C and samples were collected at the time points indicated. A: The amount of DNA-fragments transported across the cells in the apical to basolateral (A
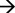
B) direction and the B
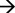
A direction was quantified by real-time PCR (qPCR) and normalized to the amount of DNA initially added to the cells and plotted against time. B: The amount of DNA-fragment left in the donor chambers after 90 min of incubation was quantified by qPCR and normalized to the amount of DNA initially added. C: All liquid in the basolateral donor chamber was collected from two wells and pooled before purification of DNA. PCR using the primers RRS SphI F and RRS SphI R was performed on the purified DNA before visualization on a 2% agarose gel to detect the full length DNA-fragment. D: After 90 min of incubation the amount of transcytosed LY was normalized to the amount of initially added LY in wells with or without addition of DNA-fragment. In A, B and D, the data shown are from one representative experiment with three replicates, showing mean +/−SD.

The time-course of DNA uptake was studied by adding 5 nM of the PCR-fragment to polarized CaCo-2 cells, either apically or basolaterally. Samples were then collected at different time points as depicted in Figure 1A. The quantity of transcytosed PCR-fragment was measured using quantitative real time PCR (qPCR) as described in the Material and Methods section and plotted as % of initially added as a function of time (Figure 1A). The amount of DNA-fragments in the acceptor chambers increased over time and after 90 minutes 0.06% of initially added was transcytosed in the apical to basolateral direction while in the basolateral to apical direction 2×10^−5^% was transported. Thus a 3×10^3^-fold difference in transport in the apical to basolateral direction was observed. Ten independent experiments were performed for the 90 minutes time point and analysed with a linear mixed-effects model, giving *p* = 0.003 (*n* = 10, number of observations  = 42). When taking samples from apical and basolateral compartments from control cells (without any additions), and adding PCR-fragment directly to these samples and incubating at 37 °C for 2 h, pronounced degradation of the fragment could be seen when running the DNA on an agarose gel (data not shown). The transcytosis of DNA-fragments as a function of time reached an apparent equilibrium after 60–90 min (Figure 1A). DNA degradation, reducing the available DNA in the donor chamber may explain this observation. We chose to use the 90 min time point as the standard time point in all the successive experiments because our interest is long term uptake and ultimately biological and health effects.

**Figure 2:**
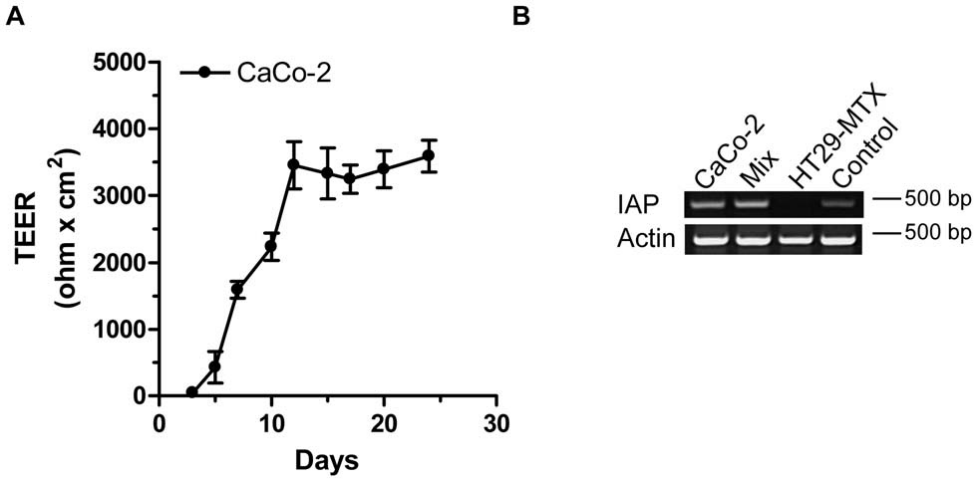
Differentiation of CaCo-2 cells. A: Trans-epithelial electric resistance (TEER) was measured on CaCo-2 cells on filters during their differentiation. Measurements were performed before change of medium. TEER (Ω x cm^2^) was plotted against time. One representative experiment is shown with mean +/−SD from nine wells. B: Intestinal alkaline phosphatase (IAP) expression at mRNA level detected by reverse transcription followed by PCR in CaCo-2 cells, CaCo-2/HT29-MTX Mix (3∶1) and HT29-MTX cells. Control is HeLa total mRNA from the Superscript III cellsdirect cDNA synthesis kit (Invitrogen). One representative experiment out of two is shown.

Formation of a polarized cell layer and differentiation of CaCo-2 cells into enterocytes was measured by detecting trans-epithelial electric resistance (TEER) and intestinal alkaline phosphatase (IAP) expression (Figure 2). IAP expression was detected in CaCo-2 cells both when cultured alone and when co-cultured with HT29-MTX cells, while IAP was not expressed in HT29-MTX cells. IAP activity is indicative of brush border formation. Monolayer and tight junction formation could also be detected upon confocal analysis of CaCo-2 cells labelled with the nuclear stain Hoechst and the actin stain phalloidin (data not shown).

The PCR-fragment used in this study was 633 bp long, giving a molecular weight of 385 kDa and contains 11 CpG motifs (Figure S1). To address whether the entire PCR-fragment (633 bp) can be transcytosed, we measured the appearance in the acceptor chambers by conventional PCR as described in the Material and Methods section. Interestingly, the full length DNA-fragment was detected in the basolateral acceptor chamber after 90 min of incubation (Figure 1C).

Lucifer Yellow dilithium salt (LY) is mainly transported across polarized cells in a paracellular fashion and was used as a marker for paracellular transport [17]. LY was added to the acceptor chambers in the absence or presence of the DNA-fragment and samples were collected after 90 min. The amount of transcytosed LY was measured as described in the Material and Methods section and plotted as % of initially added. Transport of LY was approximately two times higher in the basolateral to apical direction and was not affected by addition of DNA (Figure 1D).

### Transport mechanisms involved in the observed transport of DNA across CaCo-2 cells

The observed directionality in transport of the DNA-fragment across CaCo-2 cells indicates the involvement of transcellular vesicular transport. Membrane mediated transport can be inhibited upon incubation of cells at 4 °C. Transcytosis experiments were therefore performed on ice. Transcytosis of the DNA-fragment was then reduced approximately 100-fold (*p* = 0.03) and transport of LY was reduced approximately 4-fold (*p* = 0.05) when compared to 37 °C (Table 1). LY will, in addition to paracellular transport, follow fluid phase vesicular transport across CaCo-2 cells. This could in part explain the reduction in LY transport on ice. TEER levels increased by a factor of approximately 2 upon incubation on ice (Table 1). This could also potentially affect LY transport. Degradation of the PCR-fragment in the donor chambers was not affected by temperature (data not shown). The more effective inhibition of DNA transport than LY transport on ice suggests that DNA transport is mediated by a temperature sensitive, transcellular pathway. However, paracellular transport cannot be excluded based on this experiment only.

**Table 1:** Effect of inhibition of endocytosis on transcytosis of DNA-fragment (5 nM).

Treatment	Measured	Inhibition factor	Treated	Control	*n*	*m*	*p*
Ice	DNA-fragment, %	152	0.00015	0.023	6	35	0.03
	LY, %	4.4	0.13	0.55	7	42	0.05
	TEER, Ω×cm^2^	0.48	2528	1225	7	42	-
BafA1	DNA-fragment, %	24	0.0021	0.051	4	22	0.048
	LY, %	1.2	1.2	1.4	4	22	0.3
	TEER, Ω×cm^2^	1.3	434	546	2	18	-

Transcytosis of DNA fragments and Lucifer yellow (LY) in the apical to basolateral direction were quantified as described in Materials and Methods and tested statistically with a linear mixed-effects model. Inhibition factor is calculated as control divided by treated. The number *n* is the number of independent experiments and the number *m* is the total number of wells analysed (observations). Control for ice-treatment is 37 °C and control for bafilomycin A1 (BafA1)-treatment is with vehicle (DMSO). TEER = trans-epithelial electric resistance.

In order to elucidate if DNA-fragments are transported across CaCo-2 cells by vesicular transport, transcytosis of our DNA-fragment was measured in the absence or presence of 0.1 µM of the specific vacuolar-type H^+^-ATPase inhibitor Bafilomycin A1 (BafA1). Transport of DNA was reduced 24-fold (*p* = 0.048) in the presence of BafA1 (Table 1) while BafA1 had no effect on LY transport and TEER indicating that BafA1 did not affect tight junction integrity (Table 1). The vehicle DMSO did not affect transcytosis (data not shown) and BafA1 had no inhibitory effect on the qPCR reaction itself (analysing the linearity of a serial dilution of DNA and basolateral medium containing BafA1, data not shown). Combined, the data on ice and BafA1 demonstrates that DNA-fragments are transported across CaCo-2 cells using a transcellular, vesicle dependent pathway.

### Effect of different competitors of DNA binding on transport of DNA-fragments across CaCo-2 cells

In order to identify if DNA binding proteins are involved in the observed transport of DNA-fragments across CaCo-2 cells, transcytosis of the DNA-fragment was performed in the absence or presence of nucleic acids and anionic compounds known to bind to TLR9 (CpG ODN and GpC ODN), SR (fucoidan and heparin), NACh (heparan sulphate), nucleolin (heparin) and other DNA binding proteins (heparin, dextrane sulphate and linearized plasmid) [18]–[21]. LY and TEER were measured to monitor tight junction integrity. Linearized plasmid reduced transcytosis of the DNA-fragments across CaCo-2 cells by a factor of approximately 2.2 (*p* = 0.005, Table 2) while not affecting LY transport (Table 3). Linearized plasmid had no inhibitory effect on the qPCR itself (data not shown). Fucoidan and dextran sulphate reduced DNA transport with a factor of 2.0 and 2.9, respectively, but his reduction was not statistically significant (Table 2). Transport of DNA-fragments was, on the other hand, not affected by GpC ODN, heparin and heparan sulphate (Table 2). Surprisingly CpG ODN increased DNA and LY transport by a factor of 12 (*p* = 0.003) and 3 (*p* = 0.03), respectively, (Tables 2 and 3), while not affecting TEER (Table 4). Cytochalasin D (CytD) was used as a positive control for opening of tight junctions (Tables 3 and 4), giving an increase in LY transport by a factor of approximately 6 (p<0.001) and a reduction in TEER measurement by a factor of approximately 4.

**Table 2:** Competition for transcytosis of DNA-fragment (5 nM) by nucleic acids and anionic compounds.

Competitor	Factor	Treated, %	Control, %	*n*	*m*	*p*
pUC19 (lin) (242 nM)	2.2	0.12	0.27	3	16	0.005
CpG ODN (10 µM)	0.080	1.95	0.16	4	24	0.003
GpC ODN (10 µM)	0.82	0.048	0.039	4	*24*	*0.5*
Fucoidan (100 µg/ml)	2.0	0.13	0.26	7	*42*	*0.2*
Heparin sulphate (200 µg/ml)	0.92	0.052	0.048	5	29	0.3
Heparan sulphate (200 µg/ml)	0.79	0.21	0.17	2	12	0.6
Dextran sulphate (250 µg/ml)	2.9	0.055	0.16	4	22	0.3

Transcytosis of DNA fragments in the apical to basolateral direction with (treated) and without (control) competitor were quantified after 90 minutes of incubation as described in Materials and Methods and tested statistically with a linear mixed-effects model. Factor is calculated as control divided by treated. The number *n* is the number of independent experiments and the number *m* is the total number of wells analysed (observations). pUC19 lin = linearized pUC19 plasmid. CpG and GpC ODN = short oligonucleotides rich in CpG and GpC dinucleotide motifs, respectively.

**Table 3:** Transcytosis of Lucifer yellow (LY) in the presence of compounds affecting transcytosis of DNA-fragment.

Compound	Factor	Treated, %	Control, %	*n*	*m*	*p*
pUC19 (lin) (242 nM)	0.88	1.1	1.2	2	12	0.5473
CpG ODN (10 µM)	3.1	3.7	1.2	3	17	0.0260
Fucoidan (100 µg/ml)	0.88	1.1	1.2	2	12	0.4730
Heparin sulphate (200 µg/ml)	0.84	1.0	1.2	2	12	0.4691
Dextran sulphate (250 µg/ml)	0.66	0.27	0.41	3	17	0.4317
CytD (2.5 µg/ml)	5.7	5.5	0.97	7	40	<0.001

Transcytosis of LY in the apical to basolateral direction with (treated) and without (control) competitor was quantified after 90 minutes of incubation as described in Materials and Methods and tested statistically with a linear mixed-effects model. Factor is calculated as control divided by treated. The number *n* is the number of independent experiments and the number *m* is the total number of wells analysed (observations). pUC19 lin = linearized pUC19 plasmid. CpG ODN = short oligonucleotide rich in CG dinucleotide motifs. Control for Cytochalasin D (CytD)-treatment is with vehicle (DMSO).

**Table 4:** Trans-epithelial electric resistance (TEER) in the presence of compounds increasing Lucifer yellow (LY)-transport.

Compound	Reduction Factor	Treated, Ω×cm^2^	Dev	Control, Ω×cm^2^	Dev	*n*	*m*
CpG ODN (10 µM)	1.1	431	47	490	14	2	18
CytD (2.5 µg/ml)	4.0	164	11	655	83	2	18

TEER was measured at the end of the experiment. Deviation (dev) is calculated as the absolute value of the difference between the two experiments divided by 2. The number *n* is the number of independent experiments and number *m* is the total number of wells analysed (observations). CpG ODN = short oligonucleotide rich in CG dinucleotide motifs. Control for Cytochalasin D (CytD)-treatment is with vehicle (DMSO).

## Discussion

Here, we present evidence that dietary DNA-fragments are actively transported across the intestine by DNA binding proteins using vesicular mediated transport. Furthermore, we demonstrate that DNA binding proteins not capable of binding short single stranded ODNs with a phosphothioate backbone, fucoidan, heparin, heparan sulphate and dextran sulphate are involved in the observed transcytosis of DNA-fragments.

The observed clear directionality of DNA transport is indicative of an active transport. Our findings therefore suggest that DNA transport is following a transcellular pathway. The directionality in transport of the paracellular marker LY is opposite of that of DNA-fragments, indicating that DNA-fragments are transported across CaCo-2 cells by a different mechanism than LY. Endocytosis and vesicular transport is temperature dependent and will be inhibited at low temperatures. Our observation that transcytosis of DNA-fragments was inhibited at low temperatures could therefore be explained as inhibition of vesicular mediated uptake. Tight junctions are, on the other hand, more tightly closed when cells are incubated on ice (Table 1). This could potentially contribute to the observed inhibition of DNA transport but is contradicted by theobserved transcytosis of the full length DNA-fragment across CaCo-2 cells. The full length DNA-fragment used in our experiments is 633 bp, has a molecular weight of 385 kDa and the diameter of dsDNA is approximately 20 Å. This is well above the observed radius of 12 Å of tight junctions in CaCo-2 cells [22]. Vesicular mediated transcytosis involves acidification of the endocytic compartments by the vacuolar-type H^+^-ATPase and transport through organelles with pH in the range of 5.5–6.0 [23]. BafA1 inhibits endosomal acidification leading to a block in the maturation of early endosomes and further transport through the different endosomal compartments. Transcytosis of our DNA-fragment was inhibited upon incubation with BafA1 (Table 1). Together with the temperature dependency of DNA transport, this yields a strong indication that vesicular transport is mediating transcytosis of DNA-fragments across intestinal cells.

Vesicular mediated uptake can either occur by fluid phase uptake or by adsorptive endocytosis. Adsorptive endocytosis can, in contrast to fluid phase endocytosis, be competed out and involves receptors and proteins located at the PM. DNA binding proteins and receptors at the PM that could be involved in transcytosis are, as earlier mentioned, TLR9, MARCO, Stabilin1/2, CXCL16, NACh and nucleolin, in addition to proteoglycan-dependent macropinocytosis in combination with DNA-binding proteins released by cells [24]–[26]. To investigate the involvement of adsorptive endocytosis in transcytosis of DNA-fragments, transcytosis was performed in the presence of different ligands for DNA-binding proteins. We performed transcytosis experiments in the presence and absence of CpG and GpC containing ODNs (TLR9), fucoidan (SR-A and MARCO), heparin (SR-A, MARCO, Stabilin 1/2, CXCL16 and nucleolin), heparan sulphate (NACh) and dextran sulphate. Uptake mediated by proteoglycan-dependent macropinocytosis in combination with DNA-binding proteins released by cells is also inhibited by heparin. None of the above mentioned compounds significantly inhibited transcytosis of our DNA-fragment, excluding TLR9, SRs, NACh, nucleolin and heparin binding proteins as proteins involved in the observed transcytosis of DNA-fragments. CpG ODN but not GpC ODN, on the other hand, actually lead to an increase in uptake of DNA that might be mediated by an induction of increased fluid phase uptake; -both DNA and LY uptake was increased while the monolayer integrity, measured as TEER, was not affected (Tables 2, 3 and 4). CpG containing phosphorothioate ODNs will activate TLR9, while CpG deficient phosphorothioate ODNs will not [18], [27]. The GpC containing ODN (2006K) used in our experiments has been demonstrated to bind to TLR9 and to efficiently block CpG containing ODN (2006) mediated activation of TLR9 [18], demonstrating that both ODNs bind to TLR9. These findings suggest that activation of surface localized TLR9 induces increased fluid phase uptake and transcytosis in CaCo-2 cells, possibly by induction of ruffling and macropinocytosis.

Linearized plasmid DNA was capable of inhibiting transcytosis of our DNA-fragment. This demonstrates that the process involves adsorptive endocytosis and not fluid phase uptake. It further demonstrates that DNA-fragments destined for transcytosis binds to DNA binding proteins at the plasma membrane. The absence of inhibition by ODNs on transcytosis of our DNA-fragment suggests that the uptake mechanism of dsDNA is different from the uptake of ssODNs. A similar pattern in inhibition of DNA uptake has been demonstrated in the fibroblastoid hamster kidney cell line BHK 21, where uptake of a 146 bp dsDNA sequence could only be inhibited by linear dsDNA and dextrane sulphate and not by short ssDNA [21].

The amount of DNA-fragments left in the donor chambers after 90 min was reduced (Figure 1B). This reduction cannot be explained by transcytosis, as only a small fraction was transcytosed. The reduction of DNA-fragments in donor chambers after 90 min is most likely a result of degradation of these fragments reflecting the natural intestinal digestion process. The degradation appeared to be the same in both the apical and basolateral chamber (Figure 1B) and the difference in transcytosed PCR-fragment between the two transport directions can therefore not be explained by DNA degradation.

The primers used for qPCR amplify a fragment of 82 bp. In order to evaluate if significantly larger DNA fragments can be transcytosed, we measured the appearance of the full length PCR-fragment (633 bp) in the acceptor chambers. The full length DNA-fragment was detected in the basolateral acceptor after 90 min of incubation (Figure 1C). This indicates that even DNA-fragments of several hundred bp in length are able to pass the intestinal epithelium.

The daily diet consumed by an adult human is approximately 1 kg. The DNA content in food is typically 0.1% by weight [1], corresponding to a daily intake of approximately 1 g of DNA. We observed that approximately 0.06% of the available DNA was transcytosed across the intestine as DNA-fragments within a 90 min period. If *in vivo* uptake is 1000 times less efficient than in tissue culture this uptake of daily dietary DNA for an adult human would correspond to approximately 0.5 µg DNA or 10^10^ DNA fragments of 1000 bp. Over a lifespan and evolutionary time it is therefore reasonable to hypothesize that transcytosis of DNA-fragments can play a significant biological role possibly modulating the immune system. The hypothesis could be seen as relevant also in light of the recently launched hypothesis that viral DNA, if integrated, has a significant effect via epigenetic alterations that can be more important than insertion mutations of coding sequences [28].

We suggest that after uptake of dietary DNA-fragments from the intestine and transport into the blood system these DNA fragments bind to PRRs and exert immunomodulatory effects. Such immunomodulatory effects have recently been demonstrated for viral vaccines [29]. Dietary intake of DNA has been demonstrated to affect host immune system and play an important role in regulating the T-helper 1 (Th1)/T-helper 2 (Th2) balance towards Th1-dominant immunity [7], [30]. After oral administration of plasmid in mice a number of immune-related genes were up-regulated [31]. Additionally, nucleic acid free (NF) diets suppress T-cell response to mitogens, decrease delayed hypersensitivity to bacterial antigens and decrease resistance to *Staphylococcus aureus* and *Candida albicans* in mice [32].

The results presented in this paper advance our knowledge concerning uptake of longer dsDNA-fragments across the intestine. We demonstrate that longer dsDNA-fragments are transported across CaCo-2 cells in the apical to basolateral direction, implying involvement of active transport in this process. Mechanisms involved in the observed transcytosis of DNA-fragments are adsorptive endocytosis and vesicular mediated transport.

## Materials and Methods

### Reagents

Primers, probes, phosphorothioate CpG ODN (ISS-ODN 2006) (5'-TCGTCGTTTTGTCGTTTTGTCGTT-3') and GpC ODN (control ODN 2006K) (5'-TGCTGCTTTTGTGCTTTTGTGCTT-3') were from DNA Technology A/S (Risskov, Denmark). Fucoidan, heparin, heparan sulphate, dextran sulphate, CytD, BafA1 and LY were purchased from Sigma (St. Louis, MO, USA). Fast Red tablets were from La Roche Ltd (Basel, Switzerland). All other chemicals were from Sigma unless otherwise noted.

### Cell culture

The human IEC cell line CaCo-2 was obtained from American Type Culture Collection (ATCC, Manassas, VA, USA) and was used between passage number 43 and 53. Cells were cultured in growth medium (Dulbecco's modified Eagle's medium (DMEM) supplemented with 10% foetal bovine serum (FBS, EU standard), 2 mM L-glutamine and 1×nonessential amino acids (all from Lonza, Verviers, Belgium) at 37 °C in a humidified atmosphere containing 5% CO_2_. To obtain a monolayer of polarized and differentiated CaCo-2 cells, cells were seeded on Millicell hanging cell culture inserts with PET membranes (12 mm, 1.1 cm^2^, 0.4 µm; Millipore, MA, USA) at a density of 1×10^5^ cells/insert in growth medium supplemented with 0.5×penicillin/streptomycin solution (Lonza). Medium was changed every 2^nd^ day and the cells were used after 20–25 days of continuous cell culture. Trans-epithelial electric resistance (TEER) was monitored during growth using the Millicell^™^-ERS System (Millipore). TEER was calculated as Ω×cm^2^ of growth area and reached a plateau-level after approximately 10 days of culturing.

To verify differentiation of CaCo-2 cells into enterocytes, activity of the brush border enzyme IAP was detected using FastRed tablets according to the manufacturer's procedures. As a control we used HeLa total mRNA from the Superscript III cellsdirect cDNA synthesis kit (Invitrogen). Monolayer formation and tight junctions were detected using confocal microscopy. Shortly, CaCo-2 cells on filter inserts were washed 3 times in basolateral buffer (Hank's balanced salts solution [HBSS] w/o bicarbonate buffered with 10 mM *N*-2-hydroxyethylpiperazine-*N* ´-2-ethanesulfonic acid [HEPES]; pH = 7.4) and thereafter fixed in 4% paraformaldehyde in Sorensen buffer. Fixed cells were washed 3 times in basolateral buffer before incubation with the nuclear stain Hoechst and the actin stain Rhodamine-Phalloidin in PBS for 15 min. The filters were thereafter washed 3 times in basolateral buffer and once in ddH_2_O and mounted using Dako mounting media (Dako Denmark A/S, Glostrup, Denmark). Samples were examined and images acquired using a Zeiss LSM 510 META confocal microscope (Carl Zeiss SMT Ltd, Cambridge, UK).

### Purification of genomic DNA from plant material

Genomic DNA from the genetically modified soybean line GTS 40-3-2 (RRS) was extracted using a modified version of the Cetyl Trimethyl Ammonium Bromide (CTAB) method [34].

### PCR-fragment for transport studies

The transcytosis experiments were done using a 633 bp PCR-fragment as a mimic of dietary DNA (Figure S1). This fragment covers the 5′ end of the inserted *epsps* cassette in the genetically modified soybean line GTS 40-3-2 (RRS) starting from pos. 66 in the reverse complement of EMBL/GenBank accession no. AJ308514 extending to pos. 517 in the partially overlapping accession no. AB209952. The PCR fragment was amplified from RRS genomic DNA using the primers RRS SphI F (5′-GCATGCTTTAATTTGTTTCTATC-3′) and RRS SphI R (5′-GCATGCAGGCTGTAGCC-3′) and AmpliTaq Gold® DNA polymerase with Buffer II and 2.5 mM MgCl_2_ (Applied Biosystems, Life Technologies Corporation, Carlsbad, CA, USA). The PCR products were visualized on a 2% agarose gel and the 633 bp fragment was purified from the gel using the Wizard® SV Gel and PCR Clean-Up System (Promega, Madison, WI, USA). The PCR-fragment was quantified using a NanoDrop ND-1000 Spectrophotometer (NanoDrop Technologies, Wilmington, DE, USA).

### Transport assay

The cells were washed twice before pre-incubation at 37 °C w/o CO_2_ for 30 min in apical buffer (HBSS w/o bicarbonate buffered with 10 mM 2-*N*-morpholino-ethanesulfonic acid [MES]; pH = 6.5) and basolateral buffer (HBSS w/o bicarbonate buffered with 10 mM HEPES; pH = 7.4). After pre-incubation, the PCR-fragment was added directly to the donor chamber to give a final concentration of approximately 3×10^9^ copies/µl (corresponds to 1.9 µg/ml or 5 nM PCR-product). Final volume in both chambers was 500 µl. Samples of 15 µl were collected from the acceptor chambers at different time points. Samples of 15 µl were also collected from the original solutions. All samples were immediately incubated for 5–10 min at 95 °C to destroy DNase activity and thereafter stored at −20 °C. As a control, cells without addition of PCR-fragment were used. Amount of PCR-product in the samples was measured by qPCR and transcytosed PCR-product was calculated as % of initially added. For inhibition of vesicle mediated transport, the experiments were either performed on ice or in the presence of 0.1 µM of the specific vacuolar-type H^+^-ATPase inhibitor BafA1 (from a stock solution of 0.1 mM in DMSO) in both chambers during the pre-incubation and during the experiment. For opening of tight junctions, experiments were performed in the presence of 2.5 µg/ml of the actin polymerization inhibitor CytD (from a stock solution of 5 mg/ml in DMSO) in both chambers during the pre-incubation and during the experiment. For competition studies, cells were incubated with 10 µM ODNs, 100 µg/ml fucoidan, 200 µg/ml heparin, 200 µg/ml heparan sulphate, 250 µg/ml dextran sulphate or 242 nM linearized pUC19 plasmid (NEB, Ipswich, MA, USA) in the donor chambers during the pre-incubation and during the experiment (all compounds from stock solutions in ddH_2_O). Linearization of pUC19 plasmid was done using the restriction enzyme *Eco*RI (NEB). The integrity of the cell layer was monitored by TEER measurements at the start of pre-incubation, at the start of incubation with DNA-fragment and at the end of the experiment only in wells where no DNA-fragment was added to avoid cross contamination. Additionally, tight junction integrity was monitored using the paracellular transport marker LY. LY (from a stock in ddH_2_O) was added at the same time as the DNA-fragment to give a final concentration of 50 µg/ml and samples were collected from both chambers and the original solutions at the end of the experiment. The level of LY in the samples was measured with the FITC-filter settings (485 nm excitation and 535 nm emission wavelengths) using Victor2 Multilabel Counter (Wallac, Turku, Finland). Transport of LY was calculated as % of initially added.

### Detection of transcytosed full length PCR-fragment

Two acceptor chambers were pooled from the 90 min point (giving a sample volume of 1 ml) and DNA was purified using the Wizard® SV Gel and PCR Clean-Up System (Promega). PCR was performed on the purified DNA, using the primers RRS SphI F and RRS SphI R as described above. PCR-product was detected on a 2% agarose gel.

### qPCR

qPCR was used to quantify the amount of PCR-product transcytosed. Primers and TaqMan® probe used corresponds to the P35S screening module of annex B1 of ISO 21570:2005. The PCR reaction volume was 25 µl, containing 2 µl of sample, 0.3 µM of each primer 35s-F (5′-GCCTCTGCCGACAGTGGT-3′) and 35s-R (5′-AAGACGTGGTTGGAACGTCTTC-3′) and 0.15 µM of probe 35s-TMP (5′FAM-CAAAGATGGACCCCCACCCACG-Tamra-3′) in 1×TaqMan Universal PCR Master mix (#4304437, Applied Biosystems, Life Technologies Corporation, Carlsbad, CA, USA). Each biological replicate was analysed in duplicate on a Stratagene Mx3005P real-time cycler (Stratagene, La Jolla, CA, USA) with the amplification program: 2 min at 50 °C, 10 min at 95 °C, 50 cycles of 15 s at 95 °C and 60 s at 60 °C. DNA concentrations were calculated using a standard curve in the range of 1×10^6^–1×10^1^ copies/µl for samples from the acceptor chamber, and in the range of 1×10^10^–1×10^7^ copies/µl for samples from the donor chamber and original solutions. Standard for the donor chambers, samples from the donor chambers and original solutions were all diluted 1∶100 in ddH_2_O due to high template concentrations.

### Data analysis and statistics

To test if there were significant differences between the trancytosis fractions (z_ijk_) in the various treatments and their corresponding controls, a linear mixed-effects model including intercepts for the biological replicates, was used in order to account for the dependence structure of the data. Statistical tests were performed in the software R (http://www.r-project.org/), v. 2.15.1, using the package nlme (http://cran.r-project.org/web/packages/nlme/index.html) for mixed-effects modeling: 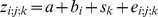, where the outcome 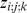 is the observed transcytosis fraction, *a* is the overall intercept, 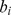 is the fixed effect of each treatment *i* = 1;2, 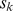 is the random effect for each experiment *k* = 1;…; n and 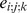 is the residual. The observation number is designated *j*. The *p*-value for the fixed effect, *b*, is reported.

In the tables the medians for both control and treatment were reported. The two technical replicates were averaged before the median was taken on the biological parallels in each experiment and then on the set of experiments. At the 90 minutes time point reported in the text, ten independent transcytosis experiments were performed, five with three biological replicates, one with two and four with one. Outliers detected using the Grubbs test [35] were removed. Fourteen data points were removed as outliers in the data set, leaving a total of 538 data points for analysis.

## Supporting Information

Figure S1 **Sequence of PCR-product used as DNA material for transcytosis experiments.** Higher case letters mark soy genomic DNA, while lower case letters mark the genetically modified insert in GTS 40-3-2 (RoundupReady) soy. CpG dinucleotide motives are in bold and the primers and probe used for quantitative real-time PCR are underlined. The sequence in part corresponds to the reverse complementary sequence reported in EMBL/GenBank accession no. AJ308514.
(PDF)

## References

[1] Forsman A, Ushameckis D, Bindra A, Yun Z, Blomberg J (2003) Uptake of amplifiable fragments of retrotransposon DNA from the human alimentary tract. Mol Genet Genomics 270: 362–368. 14556071 10.1007/s00438-003-0930-3

[2] Einspanier R, Klotz A, Kraft J, Aulrich K, Poser R, et al (2001) The fate of forage plant DNA in farm animals: a collaborative case-study investigating cattle and chicken fed recombinant plant material. European Food Research and Technology 212: 129–134.

[3] Reuter T, Aulrich K (2003) Investigations on genetically modified maize (Bt-maize) in pig nutrition: fate of feed-ingested foreign DNA in pig bodies. European Food Research and Technology 216: 185–192.

[4] Schubbert R, Renz D, Schmitz B, Doerfler W (1997) Foreign (M13) DNA ingested by mice reaches peripheral leukocytes, spleen, and liver via the intestinal wall mucosa and can be covalently linked to mouse DNA. Proceedings of the National Academy of Sciences of the United States of America 94: 961–966. 9023365 10.1073/pnas.94.3.961 PMC19622

[5] Sissener NH, Johannessen LE, Hevroy EM, Wiik-Nielsen CR, Berdal KG, et al (2010) Zebrafish (Danio rerio) as a model for investigating the safety of GM feed ingredients (soya and maize); performance, stress response and uptake of dietary DNA sequences. British Journal of Nutrition 103: 3–15. 19706208 10.1017/S0007114509991401

[6] Nielsen C, Berdal K, Bakke-McKellep A, Holst-Jensen A (2005) Dietary DNA in blood and organs of Atlantic salmon (Salmo salar L.). European Food Research and Technology 221: 1–8.

[7] Sudo N, Aiba Y, Takaki A, Tanaka K, Yu XN, et al (2000) Dietary nucleic acids promote a shift in Th1/Th2 balance toward Th1-dominant immunity. Clinical and Experimental Allergy 30: 979–987. 10848920 10.1046/j.1365-2222.2000.00811.x

[8] Wu-Pong S, Livesay V, Dvorchik B, Barr WH (1999) Oligonucleotide transport in rat and human intestine ussing chamber models. Biopharmaceutics & Drug Disposition 20: 411–416. 10951429 10.1002/1099-081x(199912)20:9<411::aid-bdd208>3.0.co;2-4

[9] Basner-Tschakarjan E, Mirmohammadsadegh A, Baer A, Hengge UR (2004) Uptake and trafficking of DNA in keratinocytes: evidence for DNA-binding proteins. Gene Therapy 11: 765–774. 14724668 10.1038/sj.gt.3302221

[10] Hemmi H, Takeuchi O, Kawai T, Kaisho T, Sato S, et al (2000) A Toll-like receptor recognizes bacterial DNA. Nature 408: 740–745. 11130078 10.1038/35047123

[11] Barton GM, Kagan JC, Medzhitov R (2006) Intracellular localization of Toll-like receptor 9 prevents recognition of self DNA but facilitates access to viral DNA. Nat Immunol 7: 49–56. 16341217 10.1038/ni1280

[12] Peiser L, Mukhopadhyay S, Gordon S (2002) Scavenger receptors in innate immunity. Current Opinion in Immunology 14: 123–128. 11790542 10.1016/s0952-7915(01)00307-7

[13] Gursel M, Gursel I, Mostowski HS, Klinman DM (2006) CXCL16 influences the nature and specificity of CpG-Induced immune activation. Journal of Immunology 177: 1575–1580. 10.4049/jimmunol.177.3.1575 16849465

[14] Leal-Pinto E, Teixeira A, Tran B, Hanss B, Klotman PE (2005) Presence of the nucleic acid channel in renal brush-border membranes: allosteric modulation by extracellular calcium. American Journal of Physiology-Renal Physiology 289: F97–F106. 15727991 10.1152/ajprenal.00196.2004

[15] Shi F, Gounko NV, Wang X, Ronken E, Hoekstra D (2007) In situ entry of oligonucleotides into brain cells can occur through a nucleic acid channel. Oligonucleotides 17: 122–133. 17461769 10.1089/oli.2007.0034

[16] Watanabe T, Hirano K, Takahashi A, Yamaguchi K, Beppu M, et al (2010) Nucleolin on the cell surface as a new molecular target for gastric cancer treatment. Biol Pharm Bull 33: 796–803. 20460757 10.1248/bpb.33.796

[17] Hidalgo IJ, Raub TJ, Borchardt RT (1989) Characterization of the human colon carcinoma cell line (Caco-2) as a model system for intestinal epithelial permeability. Gastroenterology 96: 736–749. 2914637

[18] Latz E, Schoenemeyer A, Visintin A, Fitzgerald KA, Monks BG, et al (2004) TLR9 signals after translocating from the ER to CpG DNA in the lysosome. Nat Immunol 5: 190–198. 14716310 10.1038/ni1028

[19] Hanss B, Leal-Pinto E, Bruggeman LA, Copeland TD, Klotman PE (1998) Identification and characterization of a cell membrane nucleic acid channel. Proceedings of the National Academy of Sciences of the United States of America 95: 1921–1926. 9465118 10.1073/pnas.95.4.1921 PMC19214

[20] Wittrup A, Sandgren S, Lilja J, Bratt C, Gustavsson N, et al (2007) Identification of proteins released by mammalian cells that mediate DNA internalization through proteoglycan-dependent macropinocytosis. Journal of Biological Chemistry 282: 27897–27904. 17623661 10.1074/jbc.M701611200

[21] Lehmann MJ, Sczakiel G (2005) Spontaneous uptake of biologically active recombinant DNA by mammalian cells via a selected DNA segment. Gene Therapy 12: 446–451. 15616601 10.1038/sj.gt.3302428

[22] Adson A, Raub TJ, Burton PS, Barsuhn CL, Hilgers AR, et al (1994) Quantitative approaches to delineate paracellular diffusion in cultured epithelial cell monolayers. J Pharm Sci 83: 1529–1536. 7891269 10.1002/jps.2600831103

[23] Altschuler Y, Hodson C, Milgram SL (2003) The apical compartment: trafficking pathways, regulators and scaffolding proteins. Current Opinion in Cell Biology 15: 423–429. 12892782 10.1016/s0955-0674(03)00084-x

[24] Lee J, Mo JH, Katakura K, Alkalay I, Rucker AN, et al (2006) Maintenance of colonic homeostasis by distinctive apical TLR9 signalling in intestinal epithelial cells. Nature Cell Biology 8: 1327–U1327. 17128265 10.1038/ncb1500

[25] Lee J, Mo JH, Shen C, Rucker AN, Raz E (2007) Toll-like receptor signaling in intestinal epithelial cells contributes to colonic homoeostasis. Curr Opin Gastroenterol 23: 27–31. 17133081 10.1097/MOG.0b013e3280118272

[26] Lee J, Gonzales-Navajas JM, Raz E (2008) The "polarizing-tolerizing" mechanism of intestinal epithelium: its relevance to colonic homeostasis. Semin Immunopathol 30: 3–9. 18026955 10.1007/s00281-007-0099-7

[27] Bauer S, Kirschning CJ, Hacker H, Redecke V, Hausmann S, et al (2001) Human TLR9 confers responsiveness to bacterial DNA via species-specific CpG motif recognition. Proc Natl Acad Sci U S A 98: 9237–9242. 11470918 10.1073/pnas.161293498 PMC55404

[28] Doerfler W (2012) Impact of foreign DNA integration on tumor biology and on evolution via epigenetic alterations. Epigenomics 4: 41–49. 22332657 10.2217/epi.11.111

[29] Wang H, Moon S, Wang Y, Jiang B (2012) Multiple virus infection alters rotavirus replication and expression of cytokines and Toll-like receptors in intestinal epithelial cells. Virus Res 167: 48–55. 22497974 10.1016/j.virusres.2012.04.001

[30] Sudo N, Aiba Y, Oyama N, Yu XN, Matsunaga M, et al (2004) Dietary nucleic acid and intestinal microbiota synergistically promote a shift in the Th1/Th2 balance toward Th1-skewed immunity. Int Arch Allergy Immunol 135: 132–135. 15345911 10.1159/000080655

[31] Liu JW, Cheng J (2007) Molecular mechanism of immune response induced by foreign plasmid DNA after oral administration in mice. World Journal of Gastroenterology 13: 3847–3854. 17657840 10.3748/wjg.v13.i28.3847 PMC4611218

[32] Gil A (2002) Modulation of the immune response mediated by dietary nucleotides. European Journal of Clinical Nutrition 56: S1–S4. 10.1038/sj.ejcn.1601475 12142952

[33] Incorporated C (2002) Cell Culture Protocol CLS-AN-031W.

[34] Mazzara M, Paoletti C, Puumalaainen J, Rasulo D, Van Den Eede G (2005) Event-Specific Method for the Quantitation of Maize Line NK603 Using Real-Time PCR-Validation Report and Protocol. EUR 21825 EN: JRC32103(ISBN 32192-32179-00106-X)

[35] Grubbs FE (1969) Procedures for Detecting Outlying Observations in Samples. Technometrics 11: 1–21.

