# Supplementary material for: DNA-Fragments Are Transcytosed across CaCo-2 Cells by Adsorptive Endocytosis and Vesicular Mediated Transport
Source: PLoS One. 2013 Feb 11;8(2):e56671. doi: 10.1371/journal.pone.0056671 (PMC3569430; doi:10.1371/journal.pone.0056671)
Supplement: Figure S1 — Sequence of PCR-product used as DNA material for transcytosis experiments. Higher case letters mark soy genomic DNA, while lower case letters mark the genetically modified insert in GTS 40-3-2 (RoundupReady) soy. CpG dinucleotide motives are in bold and the primers and probe used for quantitative real-time PCR are underlined. The sequence in part corresponds to the reverse complementary sequence reported in EMBL/GenBank accession no. AJ308514. (PDF) [file pone.0056671.s001.pdf]

1 GCATGCTTTA ATTTGTTTCT ATCAAATGTT TATTTTTTTT TACTAGAAAT AACTTATTGC
61 ATTTCATTCA AAATAAGATC ATACATACAG GTTAAAATAA ACATAGGGAA CCCAAAtgga
121 aaaggaaggt ggctcctaca aatgccatca ttg**cg**ataaa ggaaaggcca t**cg**ttgaaga
181 tgcctctgc**c** **g**acagtggtc ccaaagatgg acccccaccc a**cg**aggagca t**cg**tggaaaa
241 agaaga**cg**tt ccaacca**cg**t cttcaaagca agtggattga tgtgatatct ccactga**cg**t
301 aagggatga**c** **g**cacaatccc actatcctt**c** **g**caagaccct tcctctatat aaggaagttc
361 atttcatttg gagaggaca**c** **g**ctgacaagc tgactctagc agatctttca agaatggcac
421 aaattaacaa catggcacaa gggatacaaa cccttaatcc caattccaat ttccataaac
481 cccaagttcc taaatcttca agtttcttg tttttggatc taaaaaactg aaaaattcag
541 caaattctat gttggttttg aaaaaagatt caatttttat gcaaaagttt tgttccttta
601 ggatttcagc atcagtggct acagcctgca tgc
